# Investigating the Role of Anti‐Leukemia Inhibitory Factor Antibody on Cytokine Profile in a Mouse Model of Breast Cancer

**DOI:** 10.1002/cnr2.70621

**Published:** 2026-07-08

**Authors:** Seyed Mohammad Seifati, Hossein Hadinedoushan, Hossein Ansariniya, Fateme Zare

**Affiliations:** ^1^ Reproductive Immunology Research Center Shahid Sadoughi University of Medical Sciences Yazd Iran; ^2^ Department of Immunology Shahid Sadoughi University of Medical Sciences Yazd Iran

**Keywords:** active immunization, breast cancer, cytokines, leukemia inhibitory factor, tumor immunity

## Abstract

**Background:**

Leukemia inhibitory factor (LIF), a member of the IL‐6 cytokine family, is increasingly recognized as a tumor‐promoting factor in breast cancer, where it contributes to immune suppression, tumor progression, and therapy resistance. While neutralizing LIF is an attractive therapeutic approach, little is known about the systemic immunological consequences of targeting this cytokine.

**Aimsَ:**

This study aimed to evaluate the effects of active immunization against recombinant LIF protein—designed to elicit endogenous anti‐LIF antibodies—on tumor progression and systemic cytokine responses in a murine breast cancer model.

**Methods and Results:**

Female BALB/c mice were immunized with recombinant LIF protein emulsified in Freund's adjuvant to induce anti‐LIF antibodies. Tumors were subsequently established by subcutaneous injection of 4T1 breast cancer cells after antibody titers were confirmed. Serum cytokine levels (IFN‐γ, TNF‐α, IL‐6, IL‐10, IL‐4, and TGF‐β) were measured by ELISA, and tumor growth was monitored. Active immunization against LIF significantly inhibited tumor growth compared to controls. Serum analysis revealed that anti‐LIF immunization systemically suppressed tumor‐induced IL‐10 while significantly enhancing IFN‐γ levels in tumor‐bearing mice. In contrast, IL‐4 reduction was only significant in the absence of tumor challenge, and TNF‐α levels were generally reduced by the presence of the tumor regardless of immunization.

**Conclusion:**

Our findings suggest that vaccination against LIF can induce endogenous anti‐LIF antibody responses, limit tumor progression, and selectively modulate systemic cytokine profiles, particularly through suppression of tumor‐induced IL‐10 together with enhanced IFN‐γ responses in tumor‐bearing mice. These findings support further investigation of LIF‐targeted immunization as a potential immunotherapeutic strategy in breast cancer.

## 1 Introduction

Leukemia inhibitory factor (LIF) is a pleiotropic cytokine belonging to the interleukin‐6 (IL‐6) family, originally identified as a regulator of hematopoiesis [1]. Under physiological conditions, LIF is produced by immune cells such as regulatory T cells, macrophages, and dendritic cells, as well as epithelial cells, fibroblasts, and trophoblasts. It contributes to diverse processes including embryogenesis, implantation, stem cell maintenance, tissue remodeling, and immune tolerance [2]. In addition, LIF regulates inflammation by promoting anti‐inflammatory pathways and modulating the balance between Th17 and Treg differentiation [3].

In pathological contexts, LIF has been implicated in tumor progression. Overexpression of LIF and its receptor (LIFR) has been reported in breast, prostate, melanoma, and nasopharyngeal carcinoma, where it is associated with tumor growth, metastasis, and resistance to radiotherapy or chemotherapy [4, 5].

Mechanistically, LIF enhances immune evasion by promoting Treg function, skewing macrophages toward an M2 phenotype, and inducing immunosuppressive cytokines such as IL‐10. Clinical studies have shown that high levels of LIF in breast cancer tissues correlate with poor prognosis and reduced T‐cell infiltration [2, 6].

Given these observations, LIF has emerged as a promising therapeutic target in oncology. Preclinical studies using neutralizing antibodies, soluble LIFR, or small‐molecule inhibitors have demonstrated anti‐tumor efficacy in various cancer models [7, 8, 9]. While most strategies rely on passive administration of LIF‐blocking agents, an alternative approach is active immunization against LIF, which induces endogenous anti‐LIF antibodies. This vaccination‐like strategy could provide durable immune responses and reduce the need for repeated antibody administration, though its systemic immunological effects remain underexplored.

We previously reported that induction of anti‐LIF antibodies in a murine breast cancer model altered the expression of immune‐related genes at the transcriptional level [10]. However, mRNA expression alone does not capture the functional outcomes of cytokine secretion. Building on this, the present study was designed to assess serum cytokine profiles and tumor growth dynamics following active LIF immunization in 4T1 tumor‐bearing BALB/c mice. We focused on inflammatory cytokines (IFN‐γ, TNF‐α, and IL‐6) and anti‐inflammatory cytokines (IL‐4, IL‐10, and TGF‐β) to determine whether anti‐LIF immunization shifts systemic immunity toward an anti‐tumor response.

This study therefore extends our prior work by providing functional protein‐level data and integrating immune modulation with tumor outcomes. By linking LIF blockade to measurable cytokine changes and tumor inhibition, our findings highlight the immunomodulatory role of LIF and support its candidacy as a novel target for breast cancer immunotherapy.

## 2 Material and Methods

### 2.1 Ethical Statement

All animal experiments were performed in accordance with the institutional guidelines for animal care and approved by the Ethics Committee of Shahid Sadoughi University of Medical Sciences, Yazd, Iran (Ethical code: IR.SSU.AEC.1402.020). This study is reported in accordance with the ARRIVE 2.0 (Animal Research: Reporting of In Vivo Experiments) guidelines. Animals were euthanized if tumor size exceeded 2000 mm^3^, if rapid ulceration occurred, or if body weight loss exceeded 20%.

### 2.2 Animals

The experimental design was adapted from our previous study, which investigated the effects of anti‐LIF antibodies on immune‐related gene expression in a breast cancer mouse model [10]. Female BALB/c mice (6–8 weeks old, 18–22 g) were purchased from the Pasteur Institute of Iran and housed under specific pathogen‐free conditions (22°C ± 2°C, 12 h light/dark cycle, free access to food and water). Mice were randomly assigned into three groups (*n* = 5 per group):

- EI (non‐tumor immunized): received recombinant LIF immunization without tumor induction.
- EII (tumor immunized): received recombinant LIF immunization followed by tumor induction.
- Control: received PBS plus adjuvant and tumor induction.

The PBS plus adjuvant tumor‐bearing control group was specifically included to control for the nonspecific immunostimulatory effects of Freund's adjuvant, thereby allowing assessment of immune changes associated with anti‐LIF immunization.

### 2.3 Immunization Protocol

Recombinant murine LIF protein (rLIF; Sigma, USA) was emulsified in Freund's adjuvant. Mice were immunized five times: four subcutaneous injections (at 2‐week intervals) and one final intraperitoneal booster. The first injection contained 40 μg rLIF in complete Freund's adjuvant (CFA), while subsequent injections used incomplete Freund's adjuvant (IFA) with 20 μg rLIF. Control mice received PBS emulsified with the same adjuvant.

### 2.4 Measurement of Anti‐LIF Antibody Titers

Blood samples were collected from the tail vein before each immunization to monitor antibody production. Anti‐LIF antibody titers were quantified by indirect ELISA using rLIF‐coated plates (2 μg/mL). Sera were serially diluted (1:50–1:6400), and bound IgG was detected using HRP‐conjugated anti‐mouse IgG (R&D Systems, USA). The endpoint titer was defined as the highest dilution yielding an optical density (OD) above the mean OD of negative controls + 2 standard deviations.

### 2.5 Tumor Induction

After confirming an increase in anti‐LIF antibody titers, tumor induction was performed in EII and control groups. Mice were injected subcutaneously in the right flank with 1 × 10^6^ 4T1 breast cancer cells suspended in 100 μL sterile PBS. Tumor development was monitored three times per week. Tumor size was measured with a digital caliper by recording the longest diameter (length) and the shortest diameter (width) of the tumor. Tumor volume was then calculated using the standard formula: Tumor volume = (length × width × width)/2.

At the experimental endpoint, tumors were excised, and their final weight was recorded using an analytical balance.

### 2.6 Blood Collection and Serum Preparation

At 3 weeks post‐tumor induction, mice were anesthetized, and blood samples were collected by cardiac puncture (~0.8–1 mL per mouse). Blood was allowed to clot for 30 min at room temperature, then centrifuged at 3000 rpm for 15 min. Serum was separated, aliquoted, and stored at −80°C until analysis.

### 2.7 Cytokine Analysis by ELISA

Serum concentrations of IFN‐γ, TNF‐α, IL‐6, IL‐10, IL‐4, and TGF‐β were measured using commercially available mouse ELISA kits (R&D Systems, Minneapolis, MN, USA) according to the manufacturer's instructions. The catalog numbers of the kits were as follows: IFN‐γ (Cat. No. MIF00), TNF‐α (Cat. No. MTA00B), IL‐6 (Cat. No. M6000B), IL‐10 (Cat. No. M1000B), IL‐4 (Cat. No. M4000B), and TGF‐β (Cat. No. DY1679). Serum samples were analyzed in duplicate and tested without additional dilution unless otherwise specified by the assay protocol. The assays had detection limits of 5 pg/mL for IFN‐γ, 8 pg/mL for TNF‐α, 7 pg/mL for IL‐6, 4 pg/mL for IL‐10, 5 pg/mL for IL‐4, and 10 pg/mL for TGF‐β. Standard curves were generated for each cytokine using serial dilutions of the provided recombinant standards. No samples fell below the lower limit of detection for the assays used.

### 2.8 Statistical Analysis

Data were analyzed using GraphPad Prism 8.0 software. Comparisons among groups were performed using one‐way ANOVA followed by Tukey's post hoc test. Results are expressed as mean ± SEM, and *p* < 0.05 was considered statistically significant.

## 3 Result

### 3.1 Anti‐LIF Antibody Production and Tumor Growth/Weight

As previously reported in our earlier study Seifati et al., *Scientific Reports* 2024 [10], active immunization against LIF resulted in a significant increase in antibody titers, accompanied by a marked reduction in tumor growth and final tumor weight compared to controls. In the present work, we focused specifically on systemic cytokine responses in the same immunization model, and therefore tumor growth and antibody titer data are not re‐presented here to avoid redundancy.

Tumor induction was performed only after confirming elevated anti‐LIF antibody titers in immunized mice. All mice developed palpable nodules within 10 ± 2 days following injection of 4T1 cells. As previously described, tumor volumes in the immunized group (EII) increased more slowly than in the control group (*p* = 0.0005), and growth in this group eventually plateaued. These findings suggest that the presence of anti‐LIF antibodies substantially reduced tumor progression.

### 3.2 Cytokine Profiles

**FIGURE 1:**
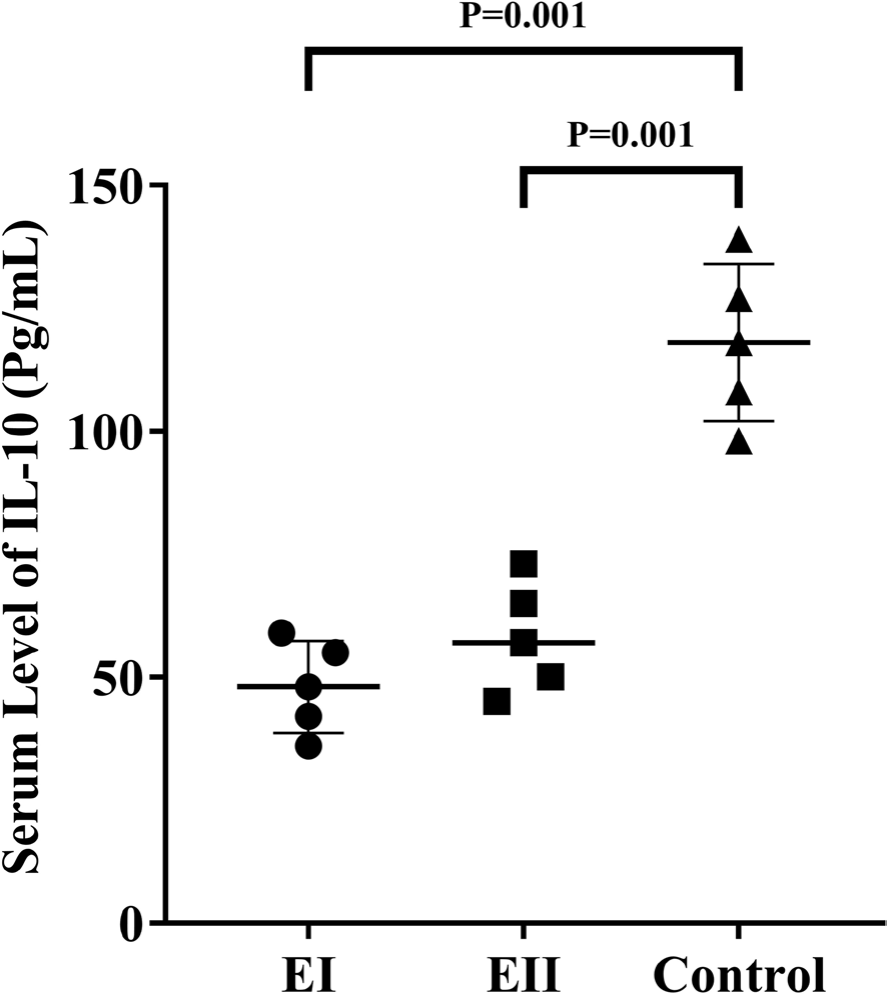
Mean serum levels of IL‐10 in the studied groups. EI: Non‐tumor immunized group; EII: Tumor‐bearing immunized group; Control: PBS plus adjuvant with tumor induction. IL‐10 levels were significantly reduced in both immunized groups compared with the Control group (EI vs. Control: *p* = 0.001; EII vs. Control: *p* = 0.001). Individual data points are shown, and data are presented as mean ± SD.

Compared with the tumor‐bearing control group, the tumor‐bearing immunized (EII) group showed significantly lower serum IL‐10 levels (*p* = 0.001). A similar reduction was also observed in the non‐tumor immunized (EI) group compared with the control group (*p* = 0.001) (Figure 1).

**FIGURE 2:**
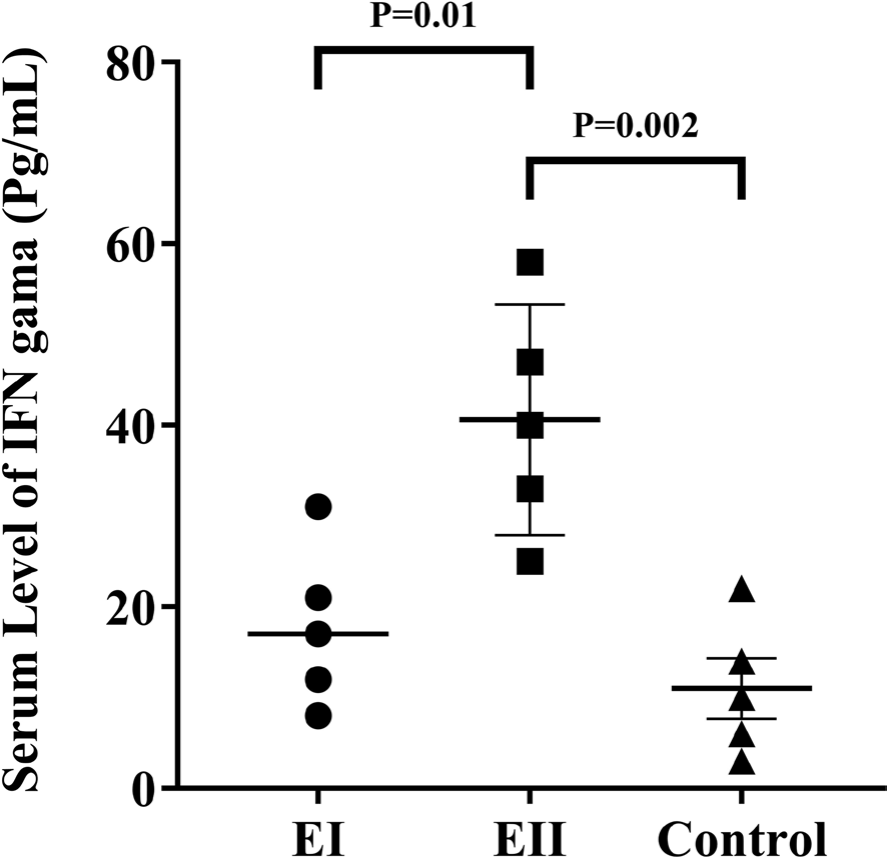
Mean serum levels of IFN‐γ in the studied groups. EI: Non‐tumor immunized group; EII: Tumor‐bearing immunized group; Control: PBS plus adjuvant with tumor induction. No significant difference in IFN‐γ levels was observed between the EI and Control groups (*p* = 0.43). In contrast, the EII group showed significantly higher IFN‐γ levels compared with the Control group (*p* = 0.002). Additionally, IFN‐γ levels in the EII group were significantly higher than those in the EI group (*p* = 0.01). Individual data points are shown, and data are presented as mean ± SD.

A significant increase in serum IFN‐γ levels was observed in the tumor‐bearing immunized (EII) group compared with the tumor‐bearing Control group (*p* = 0.002). No significant difference was detected between the EI and Control groups (*p* = 0.43), whereas IFN‐γ levels in the EII group were significantly higher than those in the EI group (*p* = 0.01) (Figure 2).

**FIGURE 3:**
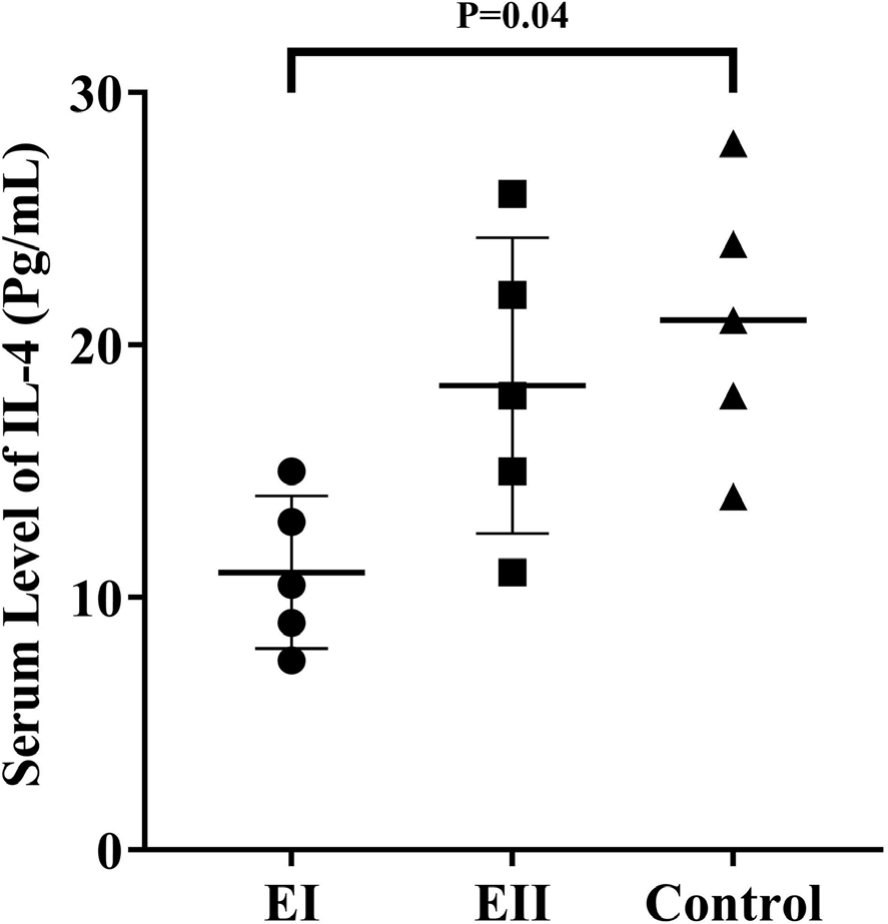
Mean serum levels of IL‐4 in the studied groups. EI: Non‐tumor immunized group; EII: Tumor‐bearing immunized group; Control: PBS plus adjuvant with tumor induction. A significant decrease in IL‐4 levels was observed in the EI group compared with the Control group (*p* = 0.04). No significant differences were observed between the EII and Control groups (*p* = 0.80) or between the EI and EII groups (*p* = 0.19). Individual data points are shown, and data are presented as mean ± SD.

No significant difference in IL‐4 levels was observed between the tumor‐bearing immunized (EII) and Control groups (*p* = 0.80). In contrast, the EI group showed significantly lower IL‐4 levels than the Control group (*p* = 0.04). No significant difference was observed between EI and EII (*p* = 0.19) (Figure 3).

**FIGURE 4:**
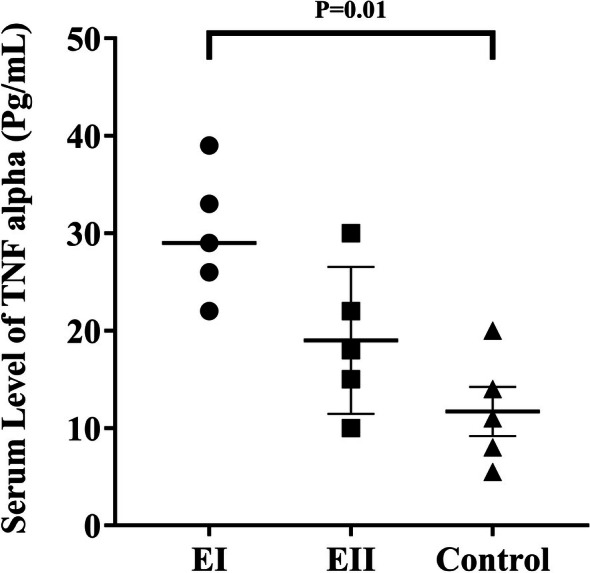
Mean serum levels of TNF‐α in the studied groups. EI: Non‐tumor immunized group; EII: Tumor‐bearing immunized group; Control: PBS plus adjuvant with tumor induction. A significant increase in TNF‐α levels was observed in the EI group compared with the Control group (*p* = 0.01), whereas no significant difference was observed between the EII and Control groups (*p* = 0.21). Individual data points are shown, and data are presented as mean ± SD.

Serum TNF‐α levels did not differ significantly between the tumor‐bearing immunized (EII) group and the tumor‐bearing Control group (*p* = 0.21). However, TNF‐α levels were significantly higher in the EI group than in the Control group (*p* = 0.01) (Figure 4).

**FIGURE 5:**
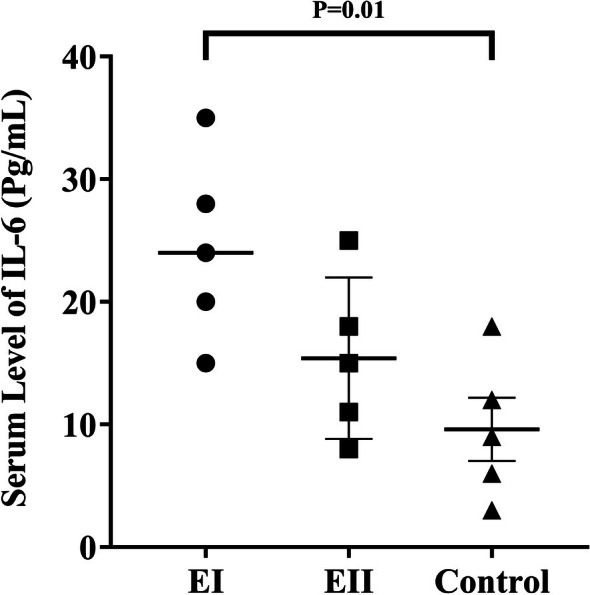
Mean serum levels of IL‐6 in the studied groups. EI: Non‐tumor immunized group; EII: Tumor‐bearing immunized group; Control: PBS plus adjuvant with tumor induction. IL‐6 levels were elevated in the immunized groups compared with the Control group; however, a statistically significant increase was observed only in the EI group following one‐way ANOVA with Tukey's multiple‐comparison test. Individual data points are shown, and data are presented as mean ± SD.

Compared with the tumor‐bearing Control group, the increase in IL‐6 levels observed in the EII group did not reach statistical significance. In contrast, IL‐6 levels were significantly higher in the EI group than in the Control group following one‐way ANOVA with Tukey's multiple‐comparison test (Figure 5).

**FIGURE 6:**
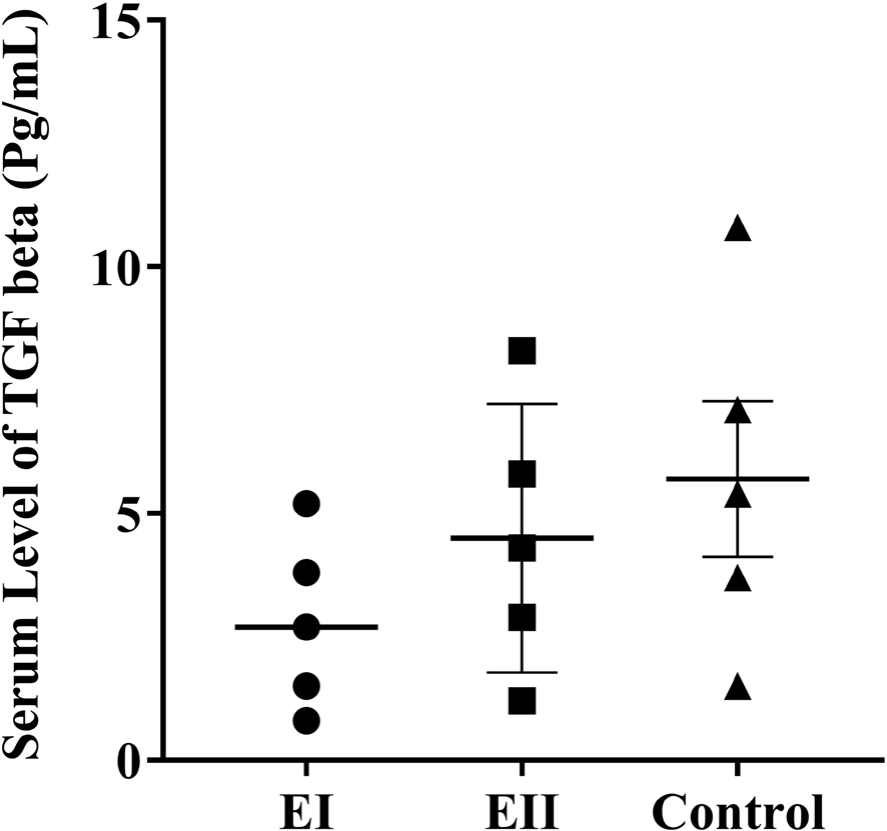
Mean serum levels of TGF‐β in the studied groups. EI: Non‐tumor immunized group; EII: Tumor‐bearing immunized group; Control: PBS plus adjuvant with tumor induction. No significant differences in TGF‐β levels were observed among the experimental groups (*p* = 0.30). Individual data points are shown, and data are presented as mean ± SD.

The measurement of TGF‐β levels indicated no significant differences among the studied groups (*p* = 0.30) (Figure 6).

## 4 Discussion

Leukemia inhibitory factor (LIF), a multifunctional cytokine of the IL‐6 family, has been implicated in diverse physiological and pathological processes, including embryogenesis, stem cell maintenance, inflammation, and immune regulation [11, 12]. LIF is expressed by immune cells, epithelial cells, fibroblasts, and tumor cells [3], and under normal conditions it contributes to tissue homeostasis and immune tolerance. In cancer, however, LIF functions as a tumor‐promoting factor, where it fosters immune evasion, drives metastasis, and contributes to therapy resistance [13, 14, 15]. Elevated LIF expression in breast tumors has been associated with tumor progression and poorer prognosis via activation of the AKT–mTOR pathway [16]. Moreover, reduced tumor‐infiltrating lymphocyte (TIL) presence is also known to predict worse clinical outcomes in breast cancer, particularly in triple‐negative and HER2‐positive subtypes [17].

While gene expression data indicate potential immune modulation, cytokine secretion reflects the ultimate effector state of immune responses and has greater translational value [10].

In line with these reports, our previous study demonstrated that immunization against LIF altered the transcriptional profile of immune‐related genes in tumor‐bearing mice. The present study extends those findings by examining systemic serum cytokine profiles at the protein level, thereby providing additional functional insight into the immunological consequences of LIF‐targeted immunization. To facilitate interpretation of the current cytokine findings, Table S1 summarizes key observations from our previously published study [10]. While gene expression data indicate potential immune modulation, cytokine secretion reflects the ultimate effector state of immune responses and has greater translational value. For example, high circulating IL‐10 and IL‐4 have been associated with poor outcomes in breast cancer patients [18], whereas elevated IFN‐γ and TNF‐α are markers of effective anti‐tumor immunity and predict improved responses to immunotherapy [19, 20].

Our results suggest that active immunization against LIF is associated with systemic suppression of tumor‐induced IL‐10 together with enhanced IFN‐γ levels in tumor‐bearing mice. Closer inspection of the data warrants caution regarding other cytokines; for instance, immunization decreased IL‐4 levels significantly only in the absence of tumor challenge (EI group), whereas in tumor‐bearing mice, IL‐4 levels did not significantly differ between the immunized and non‐immunized groups. Additionally, the presence of the tumor was associated with reduced TNF‐α levels regardless of immunization status. Nonetheless, the observed reduction in IL‐10 together with enhanced IFN‐γ levels suggests selective systemic cytokine alterations potentially associated with anti‐tumor immune activity. Importantly, although increased IFN‐γ can occur in many immunization settings, the concurrent reduction in IL‐10 together with the previously documented tumor inhibition in this model [10] supports the interpretation that these immune changes are at least partially associated with LIF‐targeted immune modulation.

Interestingly, IL‐6 levels showed a significant increase in the EI group compared with the control group, while the increase observed in the EII group did not reach statistical significance. In contrast, TGF‐β levels remained unchanged among the experimental groups. These results highlight the context‐dependent nature of LIF regulation. Previous reports showed that LIF promotes FoxP3 expression in Tregs while inhibiting IL‐6‐driven Th17 differentiation [21], suggesting that LIF blockade may differentially influence systemic inflammatory and immune‐related pathways rather than uniformly amplifying all inflammatory mediators.

Our findings are consistent with prior studies employing neutralizing antibodies or receptor antagonists against LIF. For instance, Pascual‐García et al. [7] demonstrated that LIF neutralization restored CD8+ T‐cell infiltration and improved responses to checkpoint blockade, and Liu et al. [22] reported that targeting LIF suppressed nasopharyngeal carcinoma growth and overcame radio resistance. Compared with these passive approaches, our model of active immunization against LIF offers a distinct strategy: by eliciting endogenous anti‐LIF antibodies, long‐term immune responses may be achieved without repeated antibody administration.

A critical consideration for the clinical translation of LIF‐targeted therapies is their efficacy in a therapeutic setting (i.e., administration after tumor establishment), as oncology patients present with pre‐existing tumors. In the present study, an active, prophylactic immunization protocol was utilized to establish robust endogenous antibody titers prior to tumor challenge, cleanly isolating the systemic cytokine shifts induced by the vaccine platform. Importantly, our group has previously demonstrated the feasibility of targeting LIF in a true therapeutic window using passive immunization; the administration of anti‐LIF antibodies to BALB/c mice with pre‐established 4T1 breast tumors resulted in significant tumor growth inhibition and immune modulation [13]. Thus, while passive immunization validates the therapeutic efficacy of LIF blockade post‐tumor establishment, the current study extends these findings by demonstrating that active immunization is associated with alterations in systemic cytokine balance—specifically suppressing tumor‐induced IL‐10 and enhancing IFN‐γ—offering a potentially sustained immunotherapeutic approach associated with modulation of anti‐tumor immune responses.

## 5 Limitations

At the same time, this study has several limitations. First, while we confirmed antibody titers and tumor inhibition in our earlier work, these data were not re‐presented here to avoid redundancy. Second, we did not include additional control groups such as tumor‐bearing untreated mice, non‐tumor adjuvant‐only mice, or recombinant LIF‐treated mice without adjuvant. Although the PBS plus adjuvant tumor‐bearing group was included to partially control for the immunostimulatory effects of Freund's adjuvant, we cannot completely exclude the possibility that some cytokine alterations were influenced by adjuvant‐induced immune activation. Future studies incorporating these additional controls will be important to more precisely distinguish adjuvant‐related effects from LIF‐specific immune modulation. Third, LIF secretion by 4T1 cells was not directly verified in this experiment at either the molecular or protein level, although previous studies have demonstrated high LIF expression in mouse mammary tumors [23], supporting the biological relevance of our model. Fourth, while active immunization may represent a potentially sustainable alternative to passive antibody administration, the systemic and prolonged neutralization of a pleiotropic cytokine such as LIF may carry potential risks. Under physiological conditions, LIF contributes to tissue remodeling, neuroprotection, and acute‐phase inflammatory responses. Therefore, long‐term LIF blockade could potentially impair physiological homeostasis or compromise host immune responses to acute infections. Although mice in the non‐tumor immunized group (EI) showed no obvious signs of distress or weight loss, comprehensive toxicological and infection‐challenge studies will be necessary in future investigations to fully evaluate the safety profile of this anti‐LIF vaccine strategy. Another important limitation of this study is that immune modulation was evaluated primarily through systemic serum cytokine measurements. Although these findings suggest immune alterations associated with anti‐LIF immunization, we did not directly assess tumor‐infiltrating immune populations such as CD8+ T cells, CD4+ T cells, regulatory T cells (Tregs), macrophage polarization, or myeloid‐derived suppressor cells (MDSCs). Therefore, the precise intratumoral immune mechanisms underlying the observed cytokine changes remain to be clarified. Future studies employing flow cytometry, immunohistochemistry, and tumor microenvironment profiling will be necessary to define the cellular basis of the anti‐tumor immune response induced by LIF blockade.

Despite these limitations, our results provide evidence that targeting LIF can modulate systemic cytokine balance in a manner associated with previously reported tumor inhibition. These findings reinforce the concept that LIF may function as an important regulator of tumor‐promoting immunosuppression. By reducing IL‐10 and altering selected cytokine responses, particularly IFN‐γ, anti‐LIF immunization appears to induce systemic immune changes potentially associated with anti‐tumor immune activity. Given the clinical correlations between LIF levels, cytokine signatures, and patient outcomes, our study supports further exploration of LIF‐targeted immunotherapy in breast cancer, both as a standalone strategy and in combination with immune checkpoint inhibitors.

## 6 Conclusion

In summary, this study suggests that anti‐LIF immunization is associated with systemic suppression of tumor‐induced IL‐10 together with enhanced IFN‐γ responses. These findings indicate selective systemic cytokine alterations in tumor‐bearing mice that were previously associated with reduced tumor burden in this model. Together with earlier findings on LIF‐related gene expression, our results further support the concept that LIF may contribute to tumor‐promoting immunosuppression. Unlike passive administration of neutralizing antibodies, active immunization has the potential to induce sustained endogenous anti‐LIF responses, representing a potentially novel immunotherapeutic strategy. Future studies should further validate LIF expression in tumors, characterize tumor‐infiltrating immune populations, assess predictive clinical relevance, and explore combinatorial approaches integrating LIF blockade with checkpoint inhibitors or conventional therapies. Further mechanistic and translational studies will be necessary to clarify the therapeutic potential of LIF‐targeted immunization in breast cancer.

## Author Contributions

**Seyed Mohammad Seifati:** investigation, data curation, writing – review and editing, methodology, writing – original draft. **Hossein Ansariniya:** methodology, writing – review and editing. **Fateme Zare:** investigation, methodology, writing – review and editing, conceptualization, supervision, formal analysis, project administration. **Hossein Hadinedoushan:** conceptualization, writing – review and editing.

## Funding

The authors have nothing to report.

## Conflicts of Interest

The authors declare no conflicts of interest.

## Supporting information


**Table S1:** Summary of previously reported findings from the same experimental framework used in the present study.

## Data Availability

The data supporting the findings of this study are available from the corresponding author upon reasonable request.
