# Supplementary material for: Investigating the Role of Anti‐Leukemia Inhibitory Factor Antibody on Cytokine Profile in a Mouse Model of Breast Cancer
Source: Cancer Rep (Hoboken). 2026 Jul 8;9(7):e70621. doi: 10.1002/cnr2.70621 (PMC13345775; doi:10.1002/cnr2.70621)
Supplement: Supplementary file 1 — Table S1: Summary of previously reported findings from the same experimental framework used in the present study. [file CNR2-9-e70621-s001.docx]

### **Supplementary Table S1. Summary of previously reported findings from the same experimental framework used in the present study.**

| **Parameter** | **Immunized group** | **Control group** | **Main finding** |
| --- | --- | --- | --- |
| Anti-LIF antibody response (OD450, 1:200 dilution) | ~1.2–1.5 | ~ 0.25–0.35 | Strong anti-LIF antibody induction in immunized mice |
| Tumor volume (mm³) | ~120–150 | ~500–600 | Significant reduction in tumor growth following anti-LIF immunization (p=0.0005) |
| Immune-related gene expression | Altered expression of immune-associated genes | Baseline expression profile | Previously reported transcriptional modulation associated with LIF immunization |

Data summarized from our previously published study [11] and included here to provide experimental context for the current cytokine analyses.
